# A new method for controlling an induction motor using a hybrid discretization model predictive field orientated control

**DOI:** 10.1371/journal.pone.0267459

**Published:** 2022-06-16

**Authors:** Hasan Alqaraghuli, Abdul Rashid Husain, Nik Rumzi Bin Nik Idris, Waqas Anjum, Muhammad Abbas Abbasi

**Affiliations:** 1 School of Electrical Engineering, Faculty of Engineering, Universiti Teknologi Malaysia, Johor Bahru, Malaysia; 2 Department of Electronic Engineering, Faculty of Engineering, The Islamia University of Bahawalpur, Bahawalpur, Pakistan; J.C. Bose University of Science and Technology, YMCA, INDIA, INDIA

## Abstract

The dynamic performance of the Model Predictive Control (MPC) of an Induction Motor (IM) relies on the accuracy and computational efficiency of the Discretisation Technique (DT). If the discretisation process is inaccurate or slow approximation, the MPC will exhibit high torque ripple and lower load handling capabilities. Traditionally, Euler’s method is used to discretise the MPC, which merely relies on the predictor to yield a fast, but less accurate system approximation. In contrast, Heun’s method uses a combination of predictor and corrector at alternate sampling intervals to improve the discretisation accuracy; however, the controller response becomes slow due to increased computational intensity of the algorithm. In this study, a new Hybrid Discretisation Technique (HDT) for Model Predictive Field Oriented Control (MPFOC) for IM control systems is presented to achieve robust discretisation with improved accuracy. In the proposed approach, Euler’s method is used to discretise the system at the first nine samples, followed by the predictor-corrector at the tenth sampling interval, accomplishing the desired speed and accuracy of discretisation. This newly proposed HDT in MPFOC is verified with Processor-In-Loop (PIL) for a three-phase IM with bi-directional rotation under varying load conditions. The results indicate that the IM torque ripple is reduced by up to 20%, whereas, the load handling capability is increased by up to 10%. Moreover, the controller gives 20% and 23% improvement in rise time and settling time, respectively, under high loading conditions, as compared to traditional Euler and Heun methods.

## 1 Introduction

Induction Motors (IMs) are widely employed in Variable Speed Drives (VSDs) due to their high efficiency, robust dynamic response, and low maintenance cost. However, achieving the desired control performance for IMs is highly challenging due to the factors such as, system nonlinearities, inaccurate system modelling, and varying load parameters that lead to a high steady-state torque ripple that may cause damage to the drive system. The most common vector control techniques to address these issues are field oriented control (FOC) and direct torque control (DTC) [1, 2]. The FOC uses current measurements to estimate the flux and restricts the bandwidth of the inner current controller thereby resulting in a relatively slower system response. In contrast, DTC uses current and voltage measurements to estimate the torque and flux. This improves dynamic performance such as rise-time, settling time, and overshoot; however, it increases torque ripple and requires a higher switching frequency, which may not be desirable in many applications [3, 4].

The literature proposes a diverse solution to employ advanced control methods that are capable to overcome the deficiencies that occurred by conventional DTC and FOC. several nonlinear methods have been proposed in the literature such as Sliding Mode control, Model Predictive Control. SMC can be beneficial and have a sophisticated performance when it is employed with the aforementioned conventional methods. However, the chattering phenomenon and sliding parameter optimisation consider a challenge to overcome. Nonetheless, using a command-filtered backstepping technology has a significant improvement to nonlinear system behaviour as stated in [5, 6]. The analogous method suffers from high calculation time due to complex controller design and multiple derivative equations. Subsequently, a neuroadaptive finite-time command filter is proposed in [7] to the previous problem and that solved the problem of knowing the boundary of the derivatives of the virtual signal and the chattering may occur. As a result, the method’s limitation is the high calculation time that is required to achieve the control output.

Similarly, FOC and DTC were also solved by MPC that can overcome both system non-linearities and to handle the varying load in IM [8–13]. For instance, the Predictive Torque Control (PTC) algorithm consisting of DTC and MPC technique to control the IM exhibits descent control performance under varying load parameters; however, calculating the weighting factor in this method is crucial for designing the optimised cost function [14–16]. Fuzzy logic-based decision-making is then implemented to improve computational accuracy of weighting factors; however, this approach incorporated an analytical hierarchy process (AHP) along with finite PTC (FPTC) to eliminate the search method, which made the algorithm more complex and requires higher calculation time [17]. Furthermore, a finite-state PTC (FSPTC) is proposed to reduce torque ripple that occurs in IM; however, this method requires excessive calculations that may compromise the performance of PTC [18].

The finite-control set MPC (FCS-MPC) is another method which is a combination of MPC and FOC developed to handle the model non-linearities and to handle the varying load in IM by optimising the cost function [19–21]. Similarly, MPFOC was proposed in [22, 23] to enhance the dynamic performance of IM; however, the optimisation of the cost function for optimal control performance is of prime importance in this technique. Although these conventional (DTC, FOC) and hybrid (PTC, FPTC, MPFOC) control techniques perform well in terms of IM dynamic response and load handling capability. Nevertheless, they are only focused on cost function optimisation by weighting factor selection.

The performance can further be enhanced by improving the accuracy of discretised Ordinary Differential Equations (ODE) in MPC. As the cost function optimisation is directly coupled with the discretisation method that affects the accuracy of the optimised voltage vector [24]. Numerous DTs such as Euler’s (FE) method, Heun’s (RK2) method, Runge-Kutta’s method (RK4) have been developed to represent MPC ODEs in the discrete form [25–27]. Euler’s method is the most commonly used technique among these methods due to its simplicity in approximation with a lower computational effort at the expense of modelling accuracy. Heun’s approach is adapted for system discretisation, it accurately optimises with reduced torque ripple, but the increase of computational burden slows down the system response. Similarly, the improved second-order Euler’s DT substantially reduces the torque ripple with a satisfactory prediction, but it is still not accurate due to the absence of a correction mechanism [28–30]. RK2 and RK4 methods are more accurate in terms of estimation but computationally intensive which makes their implementation a challenging task. In summary, there is a trade-off between the system dynamics response and cost-function accuracy for various DTs, but none of the aforementioned studies address both issues simultaneously. Thus, reconciling the accuracy and computational burden in model discretisation remains a challenging task.

This paper describes a new DT proposed for a MPFOC with a balance between the calculation time and the approximation accuracy to enhance the system performance. A proposed HDT consists of a predictor that is calculated using a quadratic approximation that can efficiently increase load torque and a corrector that is a new DT designed based on improved Euler’s method specifically to reduce the torque ripples generated throughout the approximation. Moreover, the new HDT will also be used also in enhancing stator flux estimator capabilities to reduce the error caused by approximation and that will yield to better overall dynamic performance. The notable merits of this paper are as follows:

- The HDT’s predictor enables the IM to handle higher torque loads whereas the conventional method was unable to handle the sudden high torque change.
- The HDT’s corrector shows a significant torque ripple reduction due to improved approximation.
- HDT substantially reduces calculation time, which contributes towords a faster system’s dynamic response.

The remaining of the paper is organised as follows: Section 2 elucidates the IM and inverter modelling, while Section 3 discusses the prediction algorithms used and the derivation of new modelling techniques. Section 4 illustrates the simulation result and comparison between conventional MPFOC with the proposed MPFOC. Finally, the last section provides a conclusion 5 that includes suggestions and enhancements.

## 2 Induction motor drive system modeling

**Fig 1:**
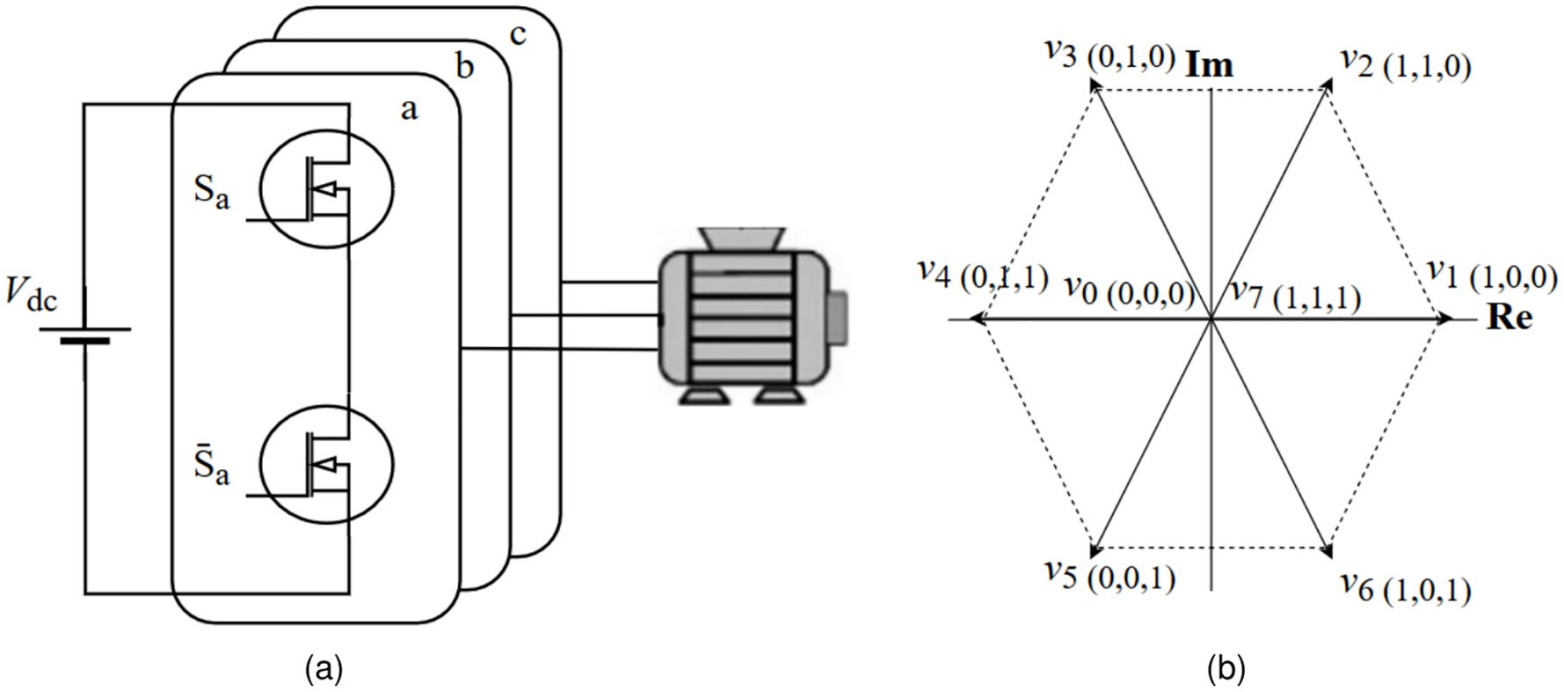
(a) Two-level three-phase VSI, and (b) voltage space vectors.

In this study, a two-level three-phase Voltage Source Inverter (VSI) is used as a power converter to drive the IM for modelling. There are two main elemnts in motor setup, Fig 1 shows the proposed inverter topology Fig 1a and its space vector modulation in Fig 1b. The switching state of the proposed inverter topology *S*, expressed in vector summation from [3], is given as:

$$\begin{array}{c} S=\frac{2}{3}\;\left(S_{a}\;+\;e^{j2\pi /3}\;S_{b}\;+\;e^{j4\pi /3}\;S_{c}\right) \end{array}$$ (1)

where, the term $S_{i},\bar{S_{i}}=1,0$ respectively, and indicates the state of on-off conditions for inverter leg for phase (a), as illustrated in Table 1, and *v* is the output voltage vector given as:

$$\begin{array}{c} v\;=\;V_{dc}\;S \end{array}$$ (2)

where *V*_*dc*_ is the DC line voltage for the squirrel-cage IM, with its rotor voltage *v*_*r*_ = 0*V*. Thus, IM could be described by the dynamics [3] given as:

$$\begin{array}{c} v_{s}\;=\;R_{s}\;i_{s}+\;\frac{d\;\psi_{s}}{dt} \end{array}$$ (3)

$$\begin{array}{c} 0\;=\;R_{r}\;i_{r}+\;\frac{d\;\psi_{r}}{dt} \end{array}$$ (4)

$$\begin{array}{c} \psi_{s}\;=\;L_{s}\;i_{s}\;+\;L_{m}\;i_{r} \end{array}$$ (5)

$$\begin{array}{c} \psi_{r}\;=\;L_{r}\;i_{r}\;+\;L_{m}\;i_{s} \end{array}$$ (6)

$$\begin{array}{c} T_{e}=\;\frac{2}{3}\;p\;Re\left(\psi_{r}^{*}\;i_{s}\right) \end{array}$$ (7)

where *v*_*s*_, *ψ*_*s*_, *ψ*_*r*_, *i*_*s*_, *i*_*r*_, *R*_*s*_, *R*_*r*_, *L*_*s*_, *L*_*r*_, *L*_*m*_, *p* and *T*_*e*_ are the stator voltage vector, stator flux, rotor flux, stator current, rotor current, stator resistance, rotor resistance, stator inductance, rotor inductance, and mutual inductance, pole pair number, and the torque, respectively. The MPC that will be formulated in this work will be based on these system dynamics.

**Table 1:** Switching states for voltage vectors.

*S*_*a*_	*S*_*b*_	*S*_*c*_	Voltage vector *V*
0	0	0	*V*_0_ = 0
1	0	0	$V_{1}=\frac{2}{3}V_{dc}$
1	1	0	$V_{2}=\frac{1}{3}V_{dc}+J\frac{\sqrt{3}}{3}V_{dc}$
0	1	0	$V_{3}=-\frac{1}{3}V_{dc}+J\frac{\sqrt{3}}{3}V_{dc}$
0	1	1	$V_{4}=-\frac{2}{3}V_{dc}$
0	0	1	$V_{5}=-\frac{1}{3}V_{dc}-J\frac{\sqrt{3}}{3}V_{dc}$
1	0	1	$V_{6}=\frac{1}{3}V_{dc}-J\frac{\sqrt{3}}{3}V_{dc}$
1	1	1	*V*_7_ = 0

## 3 Prediction algorithm

**Fig 2:**
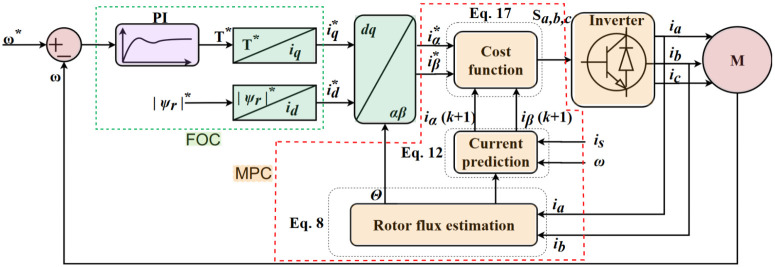
MPFOC IM drive system.

MPFOC mainly consists of two parts: 1) FOC cascade controller that handles the linear part of the model and 2) MPC that would guarantee the nonlinear system control by handling the uncertainties, and Fig 2 shows the block diagram MPFOC algorithm (green—FOC, orange—MPC). The prediction algorithm starts with stator currents prediction (*i*_*a*_, *i*_*b*_, and *i*_*c*_) along with all the calculated voltage vectors (*v*_0_, *v*_1_, …, *v*_7_). Subsequently, a cost function *g* would be evaluated for each voltage vectors where, after the evaluation only one value of voltage vector will be selected (referred to as the receding horizon prediction [3]) via minimisation function. Finally, the voltage vector *S* will produce a control signal to the Insulated-Gate Bipolar Transistors (IGBTs) for the next sample cycle. From IM dynamic equations, the stator flux estimator Eq (4) could be discretised with forward Euler’s method given by the following equation:

$$\begin{array}{c} \psi_{s}\left(k\right)=\psi_{s}\left(k-1\right)+T_{s}v_{s}\left(k\right)-T_{s}R_{s}i_{s}\left(k\right) \end{array}$$ (8)

where the stator flux at sample *k* represents the present state. In order to predict the stator flux at future step *k* + 1, it is essential to rewrite Eq (8), replacing each sample *k* for the stator flux with *k* + 1 given as:

$$\begin{array}{c} \psi_{s}^{p}\left(k+1\right)=\psi_{s}\left(k\right)+T_{s}v_{s}\left(k\right)-T_{s}R_{s}i_{s}\left(k\right). \end{array}$$ (9)

Then by substituting Eq 5 into Eq 6 the rotor flux *ψ*_*r*_(*k*) can be computed as follows:

$$\begin{array}{c} \psi_{r}\left(k\right)=\frac{L_{r}}{L_{m}}\psi_{s}\left(k\right)+(L_{m}-\frac{L_{r}.L_{s}}{L_{m}})i_{s}\left(k\right). \end{array}$$ (10)

Subsequently, the discretised predicted torque *T*^*p*^ prediction can be calculated by the following expression:

$$\begin{array}{c} T^{p}\left(k+1\right)=\;\frac{2}{3}\;p\;Re\left(\psi_{r}^{p}\left(k+1\right)\;i_{s}^{p}\left(k+1\right)\right) \end{array}$$ (11)

where this equation only considers the real part of the predicted flux and current because of the direct component *d* from the direct-quadrature framed *d* − *q*. The current required for the predicted stator can be presented as:

$$\begin{array}{c} i_{s}^{p}\left(k+1\right)=\;(1+\frac{T_{s}}{\tau_{\sigma }})i_{s}\left(k\right)+\frac{T_{s}}{T_{s}+\tau_{\sigma }}\{\frac{1}{R_{\sigma }}[(\frac{k_{r}}{\tau_{r}}-k_{r}j\omega \left(k\right))\psi_{r}\left(k\right)+v_{s}\left(k\right)]\} \end{array}$$ (12)

where, $\tau_{\sigma }=\frac{\sigma L_{s}}{R_{\sigma }};\;\tau_{s}=\frac{L_{s}}{R_{s}};\;\tau_{r}=\frac{L_{r}}{R_{r}};\;k_{s}=\frac{L_{m}}{L_{s}};\;k_{r}=\frac{L_{m}}{L_{r}};\;\sigma =1-\frac{L_{m}^{2}}{L_{s}L_{r}};\;R_{\sigma }=R_{s}+R_{r}k_{r}^{2}$ are constants. Despite the IM dynamics of Eqs (9), (11), and (12) are discretised using Euler’s technique it gives fast prediction, it suffers from low approximation accuracy which can be enhanced by using the newly designed HDT to achieve higher accuracy and better calculation time [23]. Using the proposed HDT, the control cycle for 9 samples will be implemented by using the conventional Euler’s method for robust responses; therewith, the proposed predictor-corrector will be used at the tenth control interval to improve the overall accuracy of the discretisation procedure. The newly designed discretisation technique to be used at 10th sampling interval consists of two parts; the first is called the predictor, which is a quadratic approximation, that contributes towards robust approximation whereas, the second part, which is termed as corrector, enhances the approximation accuracy by using a modified version of the enhanced Euler’s method. Thus, Eqs (9) and (12) need to be modified in accordance with the proposed approach, and it is given as:

$$\begin{array}{c} \begin{array}{ccc} P:{\hat{\psi }}_{s}^{p0}\left(k+1\right) & = & {\hat{\psi }}_{s}\left(k\right)+T_{s}v_{s}\left(k\right)-T_{s}R_{s}i_{s}\left(k\right)+...\\ & ... & \frac{T_{s}}{2}(v_{s}\left(k+1\right)+v_{s}\left(k\right)-R_{s}(i_{s}\left(k+1\right)+i_{s}\left(k\right))) \end{array} \end{array}$$ (13)

$$\begin{array}{c} C:{\hat{\psi }}_{s}^{p}\left(k+1\right)={\hat{\psi }}_{s}\left(k\right)+T_{s}f\{t\left(k\right)+\frac{T_{s}}{2},{\hat{\psi }}_{s}\left(k\right)+\frac{T_{s}}{2}f(t\left(k\right),{\hat{\psi }}_{s}\left(k\right)+\frac{T_{s}}{2}f\left(t\left(k\right),{\hat{\psi }}_{s}^{p0}\left(k+1\right)\right))\} \end{array}$$ (14)

$$\begin{array}{c} \begin{array}{ccc} P:i_{s}^{p0}\left(k+1\right) & = & i_{s}\left(k\right)+T_{s}f\left(t\left(k\right),i_{s}\left(k\right)\right)+\frac{T_{s}^{2}}{2}\frac{d}{dt}f\left(t\left(k\right),i_{s}\left(k\right)\right)\\ \text{where}\\ f(t\left(k\right),i_{s}\left(k\right)) & = & -\frac{1}{\tau_{\sigma }}i_{s}\left(k\right)+\frac{k_{r}}{\tau_{\sigma }R_{\sigma }}\left(\frac{1}{\tau_{r}}-j\omega \right)\psi_{r}\left(k\right)+\frac{1}{R_{\sigma }}v_{s}\left(k\right),\\ \frac{d}{dt}f\left(t\left(k\right),i_{s}\left(k\right)\right) & = & (\frac{1}{\tau_{\sigma }^{2}}+\frac{k_{r}L_{m}}{R_{\sigma }\tau_{\sigma }}\left(\frac{1}{\tau_{r}}-j\omega \right))i_{s}\left(k\right)+\frac{k_{r}}{R_{\sigma }\tau_{\sigma }}\left(\frac{1}{\tau_{r}}-j\omega \right)...\\ & ... & \left(L_{m}+\frac{1}{\tau_{\sigma }}\right)\psi_{r}\left(k\right)+\frac{1}{R_{\sigma }\tau_{\sigma }}v_{s}\left(k\right)+\frac{1}{T_{s}}\left(v_{s}\left(k+1\right)+v_{s}\left(k\right)\right) \end{array} \end{array}$$ (15)

$$\begin{array}{c} C:i_{s}^{p}\left(k+1\right)=i_{s}\left(k\right)+T_{s}f\{t\left(k\right)+\frac{T_{s}}{2},i_{s}\left(k\right)+\frac{T_{s}}{2}f(t\left(k\right),i_{s}\left(k\right)+\frac{T_{s}}{2}f\left(t\left(k\right),i_{s}^{p0}\left(k+1\right)\right))\} \end{array}$$ (16)

where Eq (13) represents the predictor part of the modified technique for stator flux prediction, and superscript (^0^) represents the first stage of prediction, Eq (14) which is termed as ‘corrector’ gives the correction step of for stator flux prediction. Likewise, Eqs (15) and (16) represent the predictor and corrector steps of modified DT for stator current prediction, respectively. Since the proposed HD combines both methods of Euler and a modified discretisation algorithm to form a HD algorithm based on Eqs (8) and (12) (Euler’s method), along with Eqs (13)–(16) (modified prediction and correction algorithm), this algorithm is designed to alternate between Euler and modified DT in the prescribed sequence in order to compensate for the inaccuracies arising from the use of Euler’s method to reduce torque ripple and increase load torque. The proposed algorithm minimises the cost function hence contributing towards the effective selection of voltage vectors. The cost function to be optimised is given as:

$$\begin{array}{c} g=\sum_{h=q}^{N}\left\{\right|i_{\alpha }^{*}-i_{\alpha }\left(k+h\right)|+|i_{\beta }^{*}-i_{\beta }\left(k+h\right)|+\lambda |F_{s}\left(k+1\right)-F_{s}\left(k\right)\left|+I_{m}\left(k+h\right)\right\} \end{array}$$ (17)

$$\begin{array}{c} I_{m}=\{\begin{array}{cc} 0 & \text{if}\;|i\left(k+h\right)|\leq |i_{m}ax|\\ \lambda \gg 0 & \text{if}\;|i\left(k+h\right)|>|i_{m}ax| \end{array} \end{array}$$ (18)

where *i*_*m*_ *ax* is the max current to be chosen, *F*_*s*_ is presenting the switching frequency of IGBT, and *q* represents the horizon of the prediction which is equal to one in this work. Since VSI has seven switching states in this system (*j* = 0, …, 6), the cost function would compute these seven switching vectors in each iteration for every sample of the time, and the minimal voltage vector would be chosen as a reference to the IGBTs. Algorithm 1 describes the controller steps in order to solve the MPFOC that summarises the execution of MPFOC; the stator current is measured along with rotor speed, after which the rotor and stator flux are estimated. Subsequently, the predicted stator flux vector is calculated along with the stator current prediction.

**Algorithm 1** Execution of MPFOC

1: parameter{*T*_*s*, *K*_*p*, *K*_*i*, *J*, *p*, *L*_*m*, *L*_*s*, *L*_*r*, *R*_*s*, *R*_*r*, *T*_*nom*, *phi*_*nom*, *V*_*dc*}

2: *int k* = 1        ⊳ Define discrete sample

3: *input omega*;       ⊳ measure *ω*

4: *input i*_*s*;          ⊳ measure *i*_*s*_

5: *estimate phi*_*s*;       ⊳ *ψ*_*s*_ estimation Eq 8

6: *estimate phi*_*r*;       ⊳ *ψ*_*r*_ estimation Eq 10

7: **for** *i* = 0:1:6, **do**

8:  {*prediction phi*_*s*;   ⊳ prediction for $\psi_{s}^{p}$ Eqs 13 and 14

9:  *prediction i*_*s*;     ⊳ prediction for $i_{s}^{p}$ Eqs 15 and 16

10:  $g=\sum_{h=q}^{N}\left\{\right|i_{\alpha }^{*}-i_{\alpha }\left(k+h\right)|+|i_{\beta }^{*}-i_{\beta }\left(k+h\right)|+\lambda |F_{s}\left(k+1\right)-F_{s}\left(k\right)\left|+I_{m}\left(k+h\right)\right\}$     ⊳ cost function

11: **end for**

12: *int opt* = *min*(*g*);

13: *S*_*a* = *s*(*opt*, 1)       ⊳ find inverter switching states

14: *S*_*b* = *s*(*opt*, 2)

15: *S*_*c* = *s*(*opt*, 3)

16: **return** *S*_*a*, *S*_*b*, *S*_*c*     ⊳ Return voltage vector states

## 4 PIL simulation’s results and discussion

**Fig 3:**
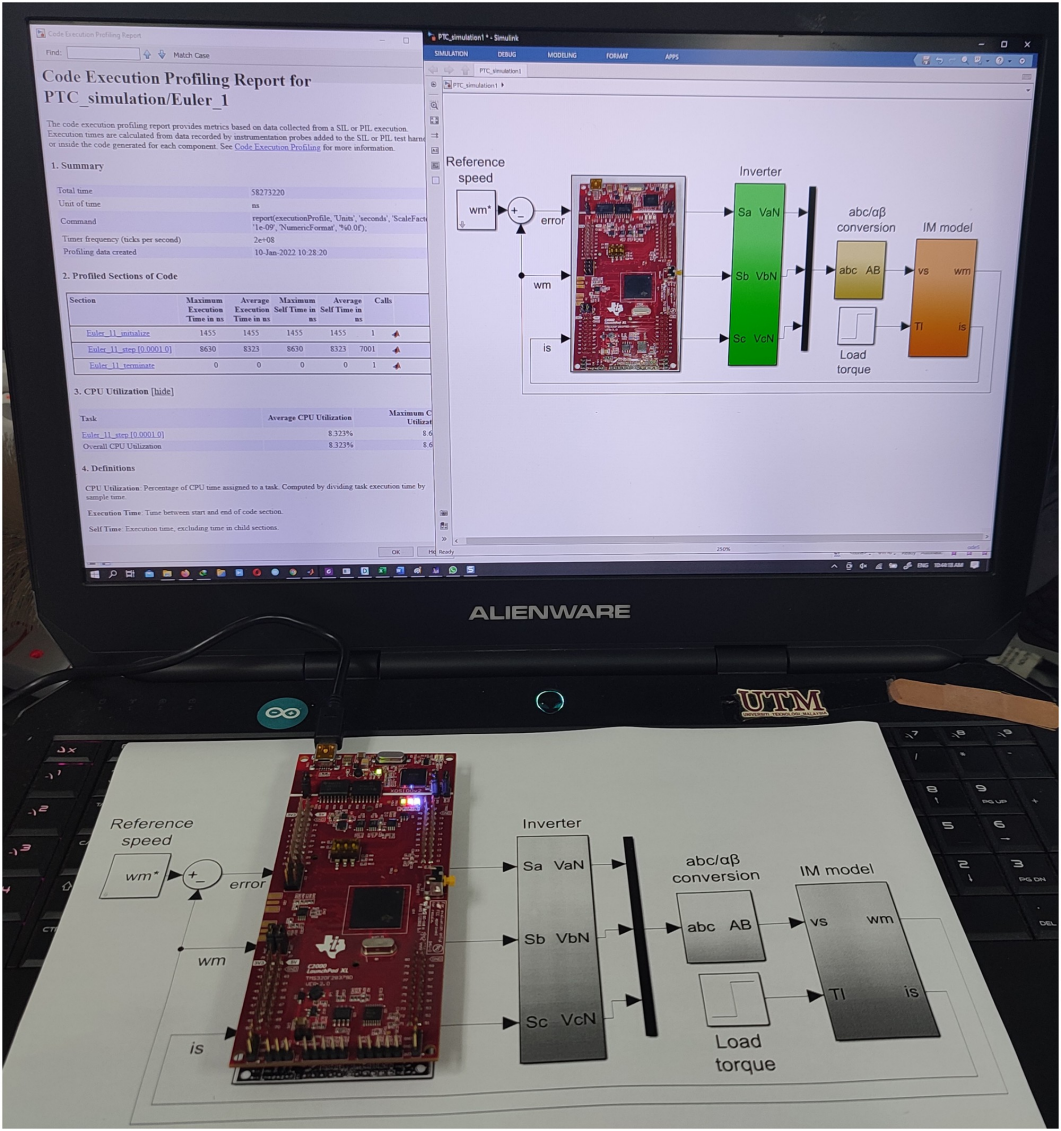
Processor-In-Loop hardware setup.

**Fig 4:**
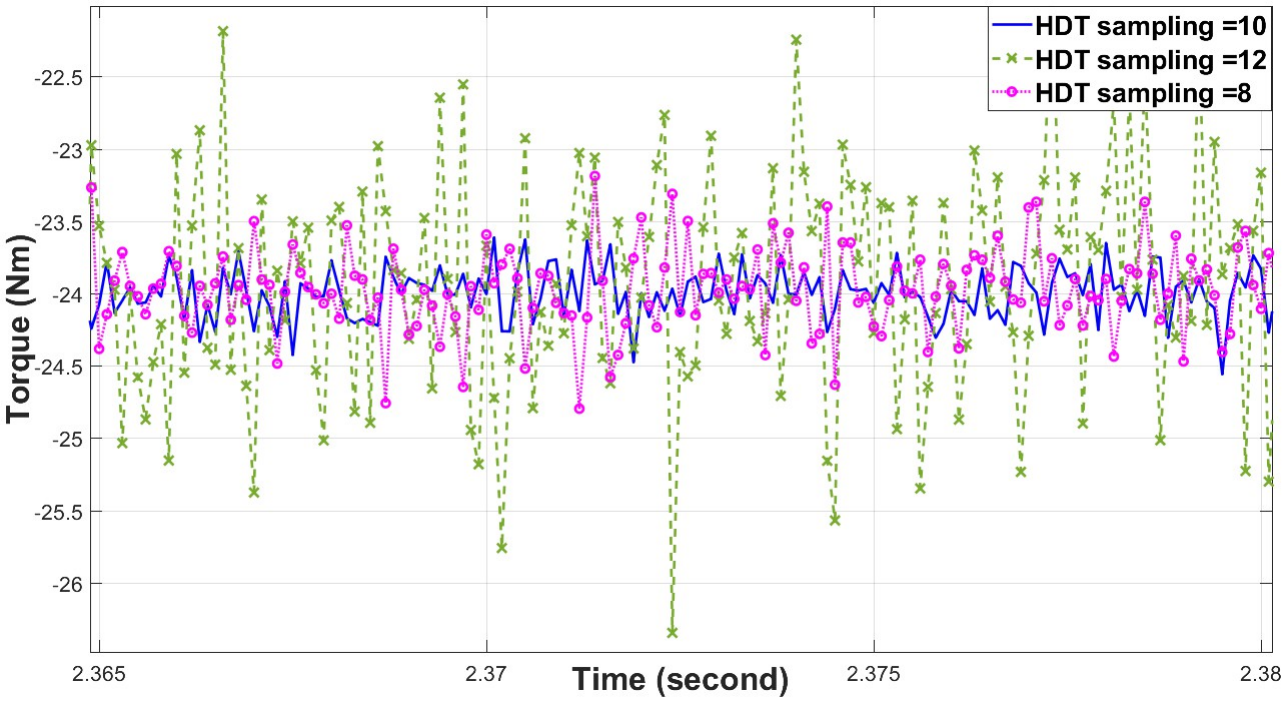
Torque ripple at 20 Nm load for HDT with three different sampling.

The verification of the proposed method is employed by using Processor-In-Loop with TMS320F28379D that can show the stability and feasibility of the proposed method Fig 3. The proposed MPC is demonstrated by PIL IM using Matlab/Simulink (version 2021b, Mathworks, USA) in order to illustrate the significance of the design. In this study, analysis of the PIL verification results is compared with those of previous studies that utilised Euler’s and Heun’s MPCs, respectively. Detailed simulation parameters for the simulation are summarised in Table 2, with the sampling frequency set at 25 kHz (calculations are performed using a 200Mhz microcontroller). An alternating sampling time of ten samples is used to depict the numerical significance of the HDT. Thus, Euler will be run nine times and HDT once every 10 samples interval. Fig 4 presents different alternating samples (8, 10, and 12) arranged to minimise torque ripple and increase torque load. In order to avoid compromising measurement quality or calculation time, it is highly important to select alternate samples carefully. Due to this circumstance, if HDT is not implemented for at least on 10th samples, the calculation time will be greater, and the processor will be involved in a longer computation process leading to a lower degree of accuracy. In comparison, if HDT is employed for more than ten samples, its accuracy will be reduced. As a final step, the best alternating sampling solution is determined by an offline binary search method.

**Table 2:** Induction motor parameters.

Parameter	Value	Parameter	Value
DC link voltage/*V*	520	*L*_*r*_/*mH*	175
*R*_*s*_/Ω	1.2	*p*	1
*R*_*r*_/Ω	1	*ω*_*nom*_/(*rad*/*sec*)	150
*L*_*m*_/*mH*	170	*T*_*nom*_/(*Nm*)	20
*L*_*s*_/*mH*	175	*J*/(*kg*.*m*^2^)	0.062

**Fig 5:**
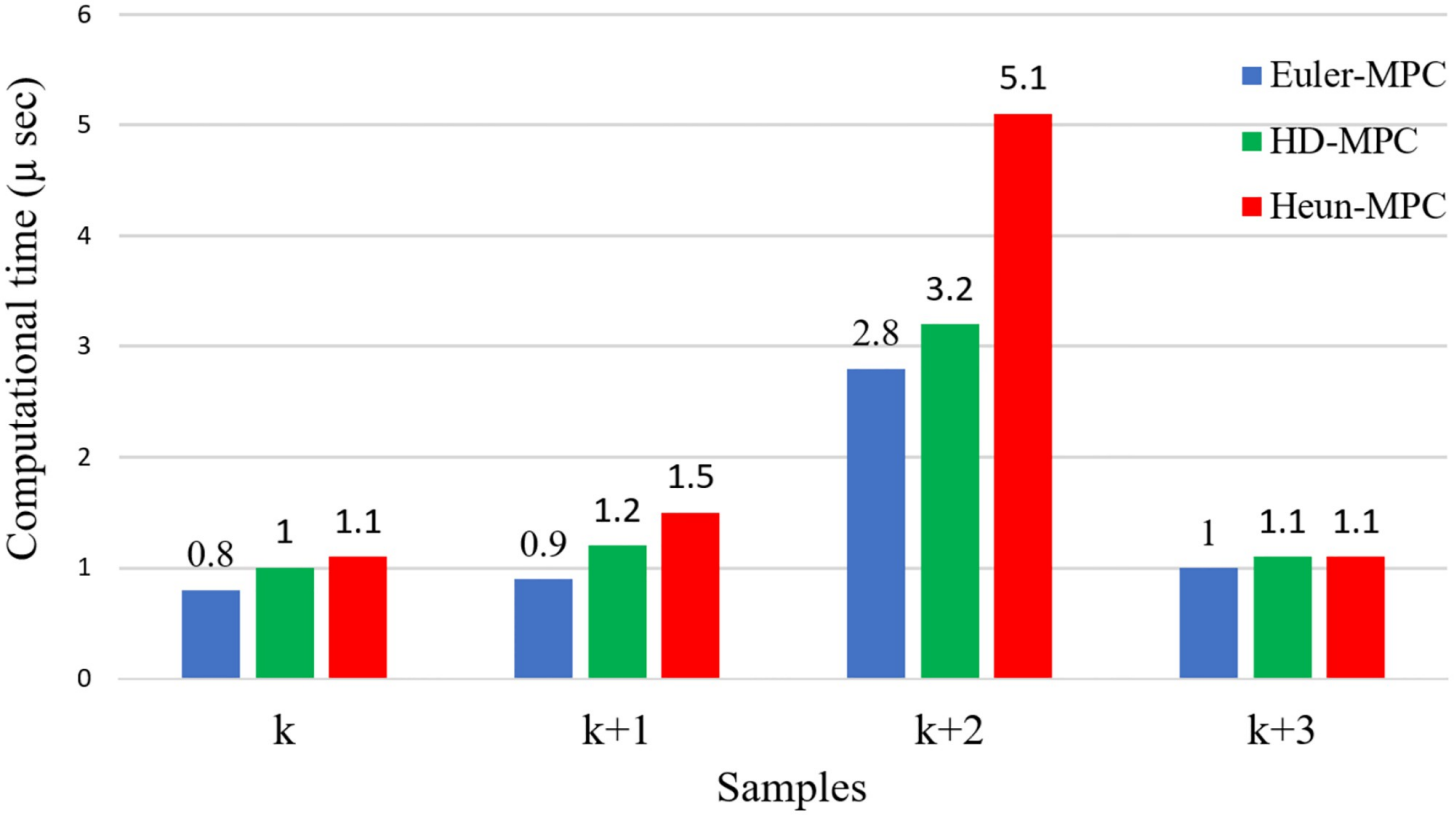
Computational time for each sample of MPFOC algorithm in Euler’s, Heun’s, and HDT’s MPCs.

**Fig 6:**
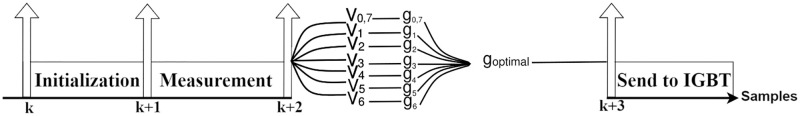
Timing diagram for MPC algorithms.

Algorithm 1 is used in this study can elucidate the steps of MPC execution, and Fig 5 shows the benchmark of computation time for Euler’s, Heun’s, and the HDT MPFOC, respectively. At the first sample *k*, the initialisation of parameters is completed. With regard to the second sample *k* + 1, the stator current and angular velocity are observed along with measurements of stator and rotor flux. Subsequently, at the time sampling *k* + 2, the seven voltage vectors are represented in this step along with predictions for the stator current, stator flux, and torque alongside the evaluation of the cost function, which is the most time-consuming step. Finally, at the last time sampling *k* + 3, the optimal voltage vector is sent to the inverter IGBT switches (*S*_*a*_, *S*_*b*_, and *S*_*c*_), as shown in the timing diagram of Fig 5 and the calculation time chart Fig 6. As the execution time of the conventional Euler’s MPC and the proposed method is nearly equal, the proposed method will not consume any more processing time. Table 3 illustrates the PIL verification for Euerl’s, Heun’s, and HDT’s methods and it is clear that the HDT calculations are considerably faster than if not using RK2 alone and it has less torque ripple and better load handling.

**Table 3:** PIL verification results.

Discretisation method	Euler’s method [19–23]	Heun’s method [25–27]	HDT’s method
Execution time (ms) for 1 second	641.260	843.755	779.988
Processor Usage (%)	64	82	75
Load torque error @ 20 Nm (%)	14.53	8.32	10.9
Speed error (%)	44.2	18.36	20
Ripple (%)	8.5	6.3	7
Maximum load torque handling capability @ 20 Nm	19.82	23.16	22.73

**Fig 7:**
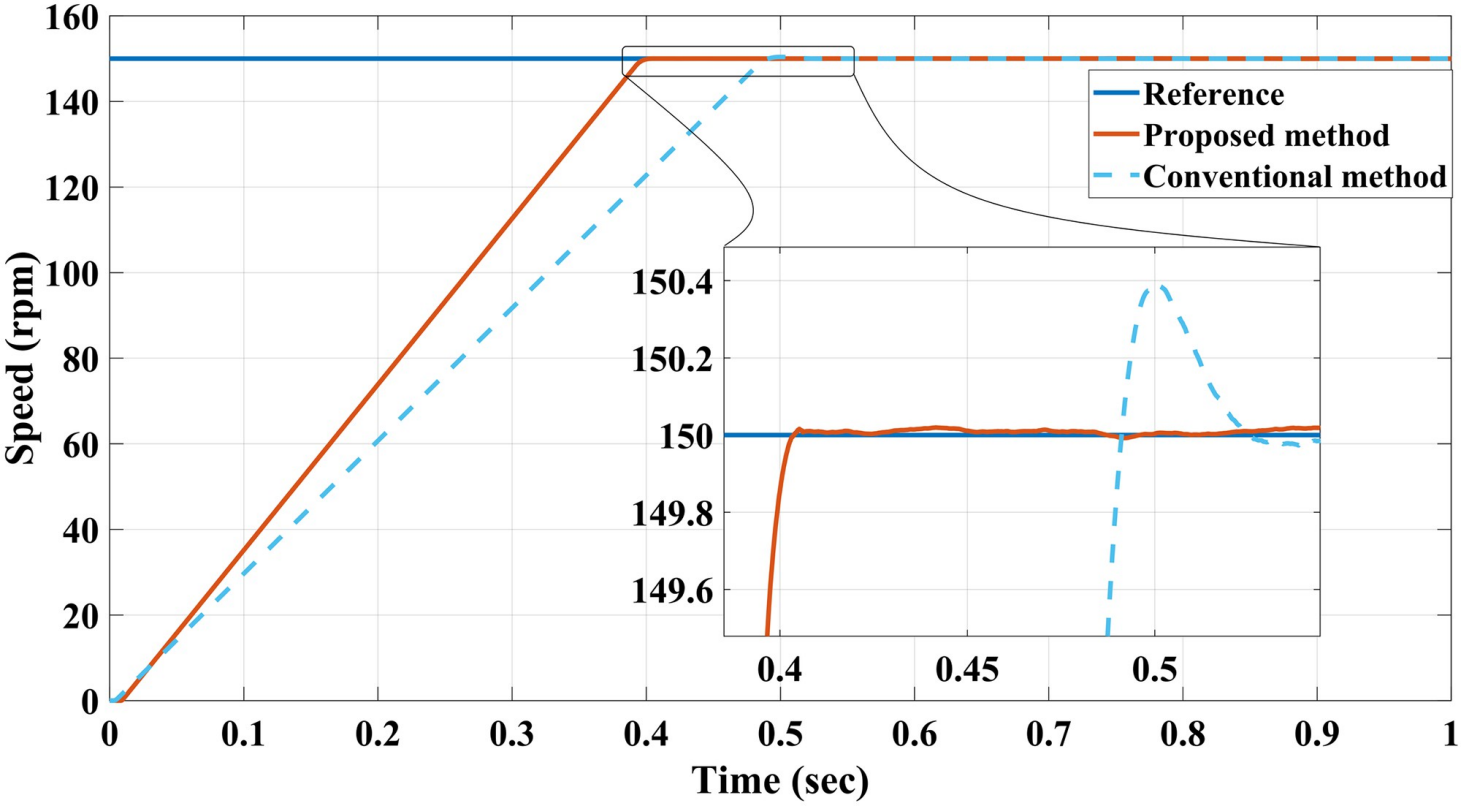
Speed response of IM at no load condition for both Euler’s MPC and HDT’s MPC.

**Fig 8:**
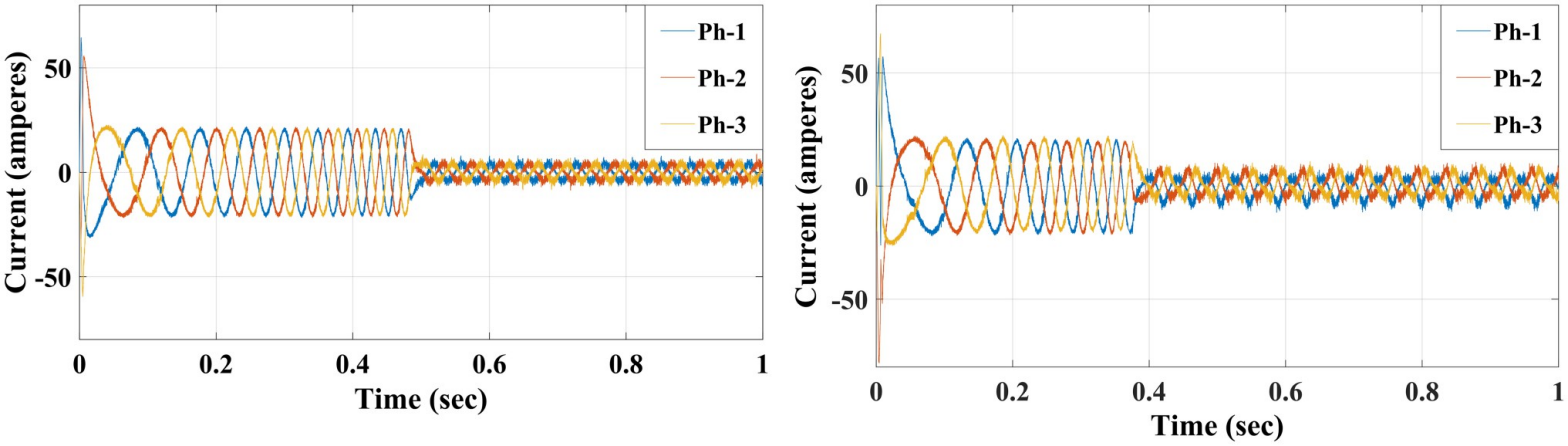
Current response of IM at no load condition for both Euler’s MPC (right) and HDT’s MPC (left).

**Fig 9:**
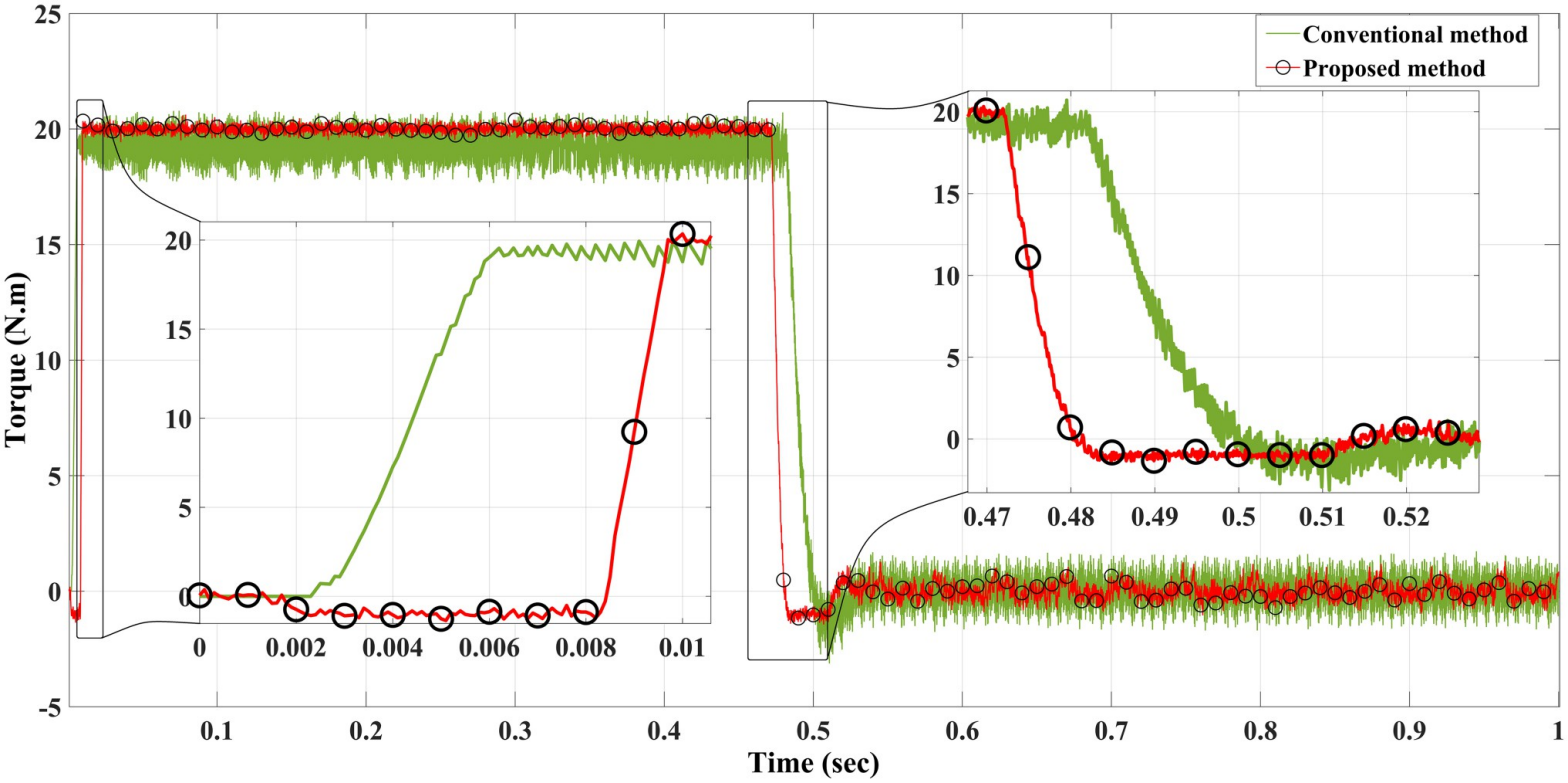
Torque response of the motor at no load for Euler’s MPC and HDT’s MPC.

**Case I**: Figs 7 and 8 illustrate the response characteristics of the conventional and proposed MPFOCs over a speed range of 150 rpm under ideal circumstances with no torque load (0 Nm). Using the method described, the transient response is improved by 12% and the settling time is shortened by 23% without overshoot. In spite of the fact that there is a delay at the beginning of the system (as explained by Eqs 13–16), this setback does not adversely affect its performance. Due to the additional term in the current prediction, the starting current of the proposed method remains higher than the conventional method; conversely, the proposed method performs similarly to the conventional method without compromising torque performance. Fig 9 shows the torque generated by both methods during the motor startup. In comparison with the conventional method, the proposed MPFOC displays improved dynamics as it provides faster response and less torque ripple during the one-step delay caused by HDT without adversely affecting the system’s performance. In summary, the proposed method enhanced the steady-state performance by 53.7% as compared to the previous method.

**Fig 10:**
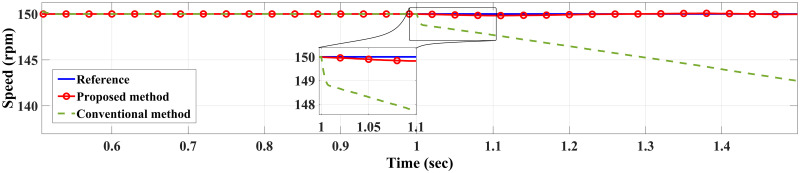
Speed response of IM at 20 Nm load for Euler’s MPC and HDT’s MPC.

**Fig 11:**
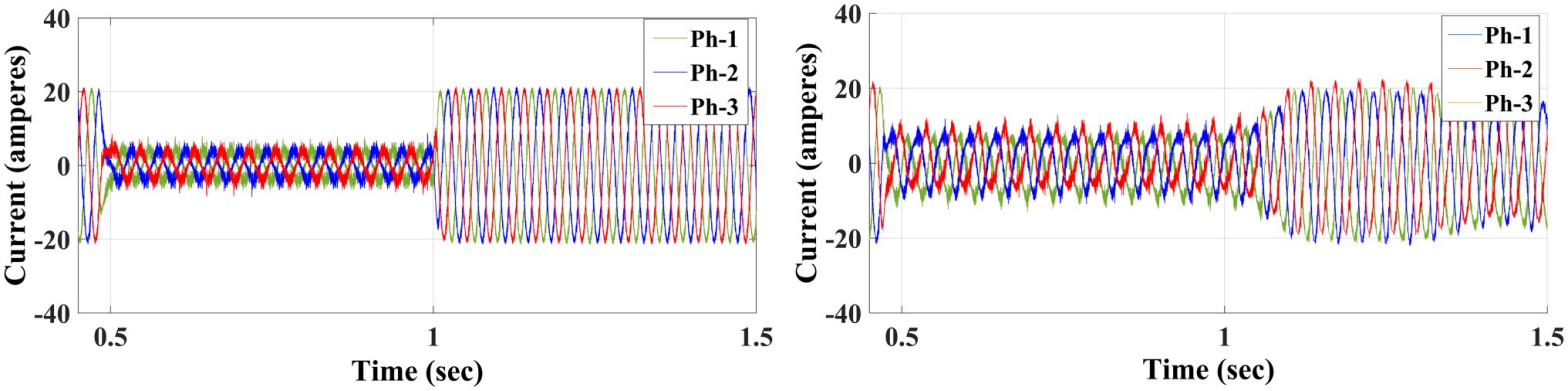
Current response of the motor at 20 Nm load for both Euler’s MPC (left) and HDT’s MPC (right).

**Fig 12:**
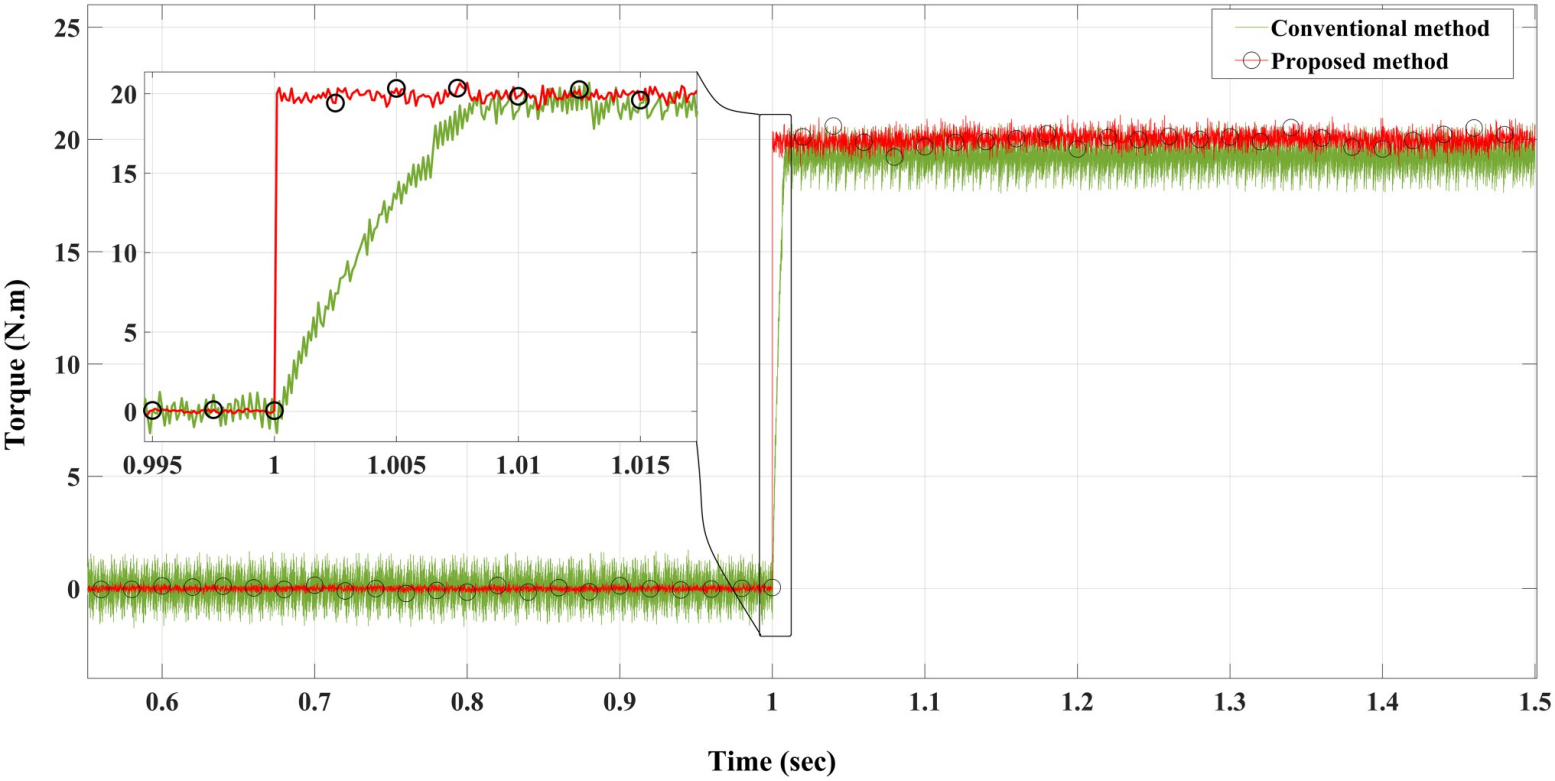
Torque response of the motor at 20 Nm load for Euler’s MPC and HDT’s MPC.

Case II: Figs 10 and 11 depict the scenario when a load with 20 Nm is applied at *t* = 1 from the steady-state speed of 150 rpm. It can be seen that the motor using the conventional method became unable to withstand the high torque due to a lack of model accuracy that is brought about by Euler’s discretisation. On the other hand, the algorithm motor controller proposed in this study handled the applied torque to the system with minor fluctuations in steady-state. Interestingly, the current drawn in the steady-state of the proposed method (10 A) is just a bit higher than that of the conventional method (8 A), which likely accounts for the fact that the proposed method is able to overcome the extremely high torque. Therefore, the conventional method torque tends to fluctuate between 20.2 Nm and 18.5 Nm, while the proposed method fluctuates between 20.4 Nm and 19 Nm as illustrated in Fig 12.

**Fig 13:**
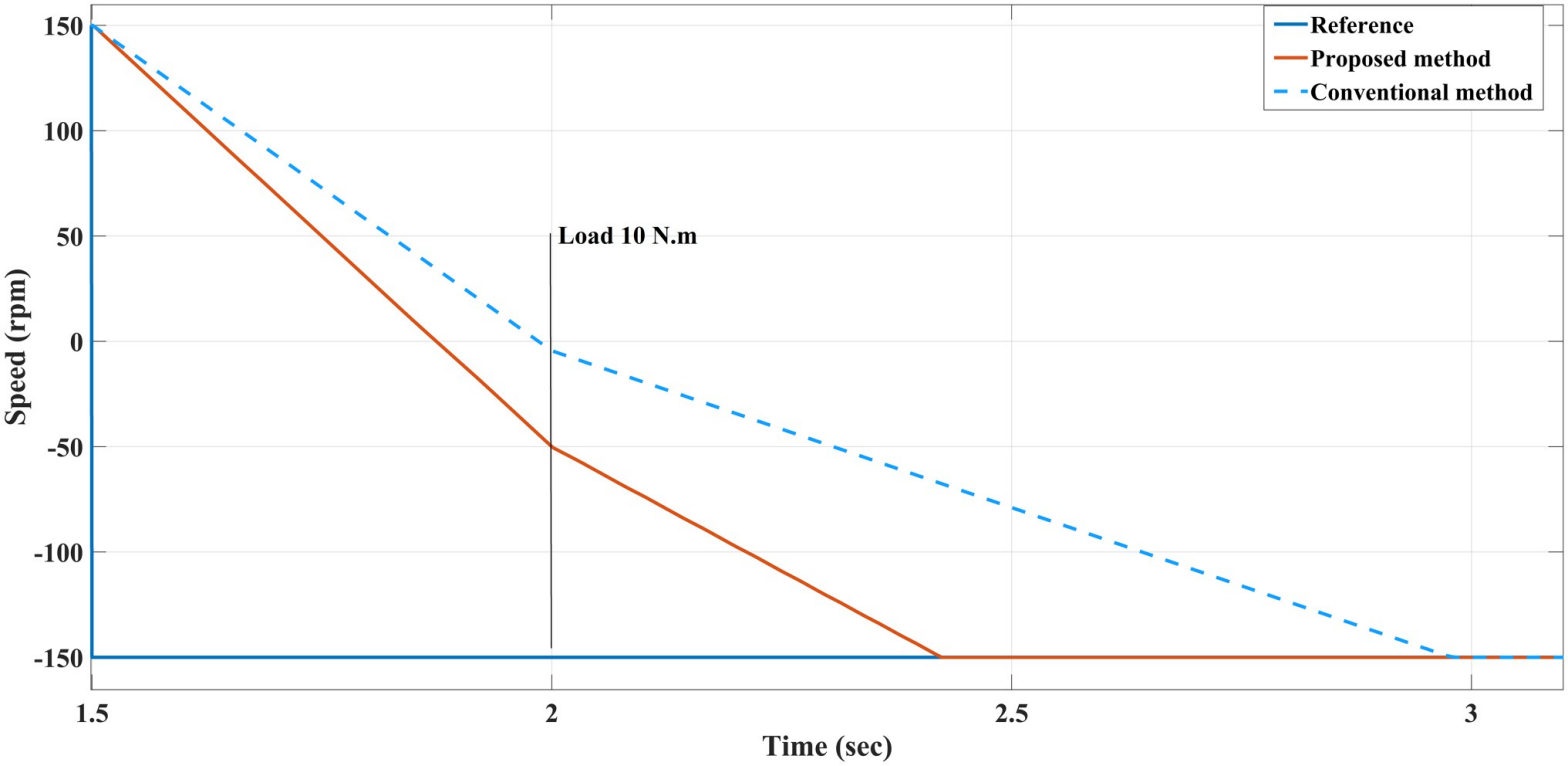
Speed response of the motor at 10 Nm reverse load CCW for Euler’s MPC and HDT’s MPC.

**Case III**: With a load of 10 Nm applied at *t* = 2 sec in counterclockwise (CCW) rotation, the conventional method achieves the steady-state at a lower torque (≈ 25 Nm) along with the proposed method. Although both motors achieve a steady-state condition, the proposed method shows a faster rise time than the conventional method, as shown in Fig 13. Both methods, however, produce similar results when it comes to current generated and torque generated.

**Fig 14:**
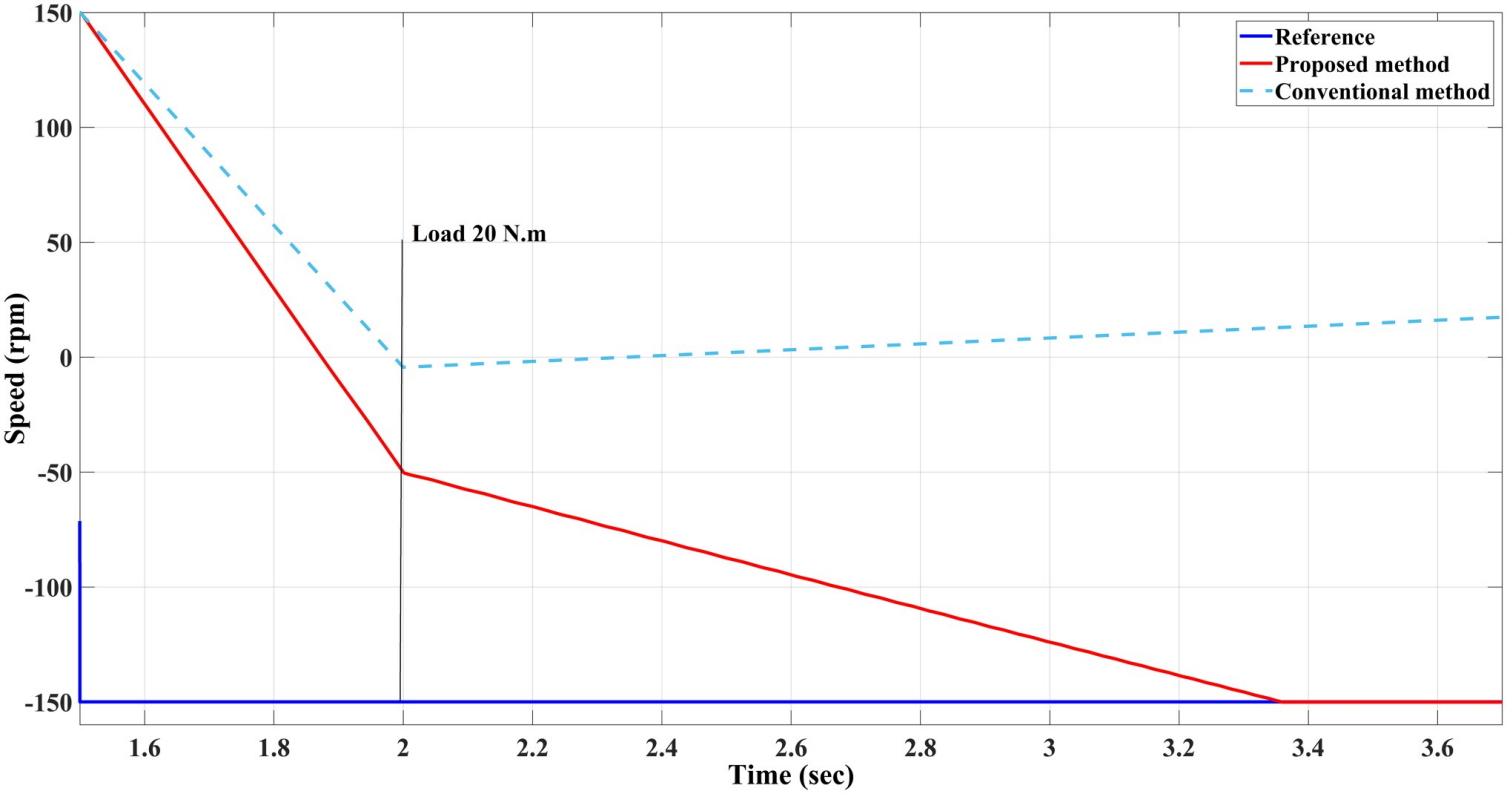
Speed response of the motor at 20 Nm reverse load CCW for Euler’s MPC and HDT’s MPC.

**Fig 15:**
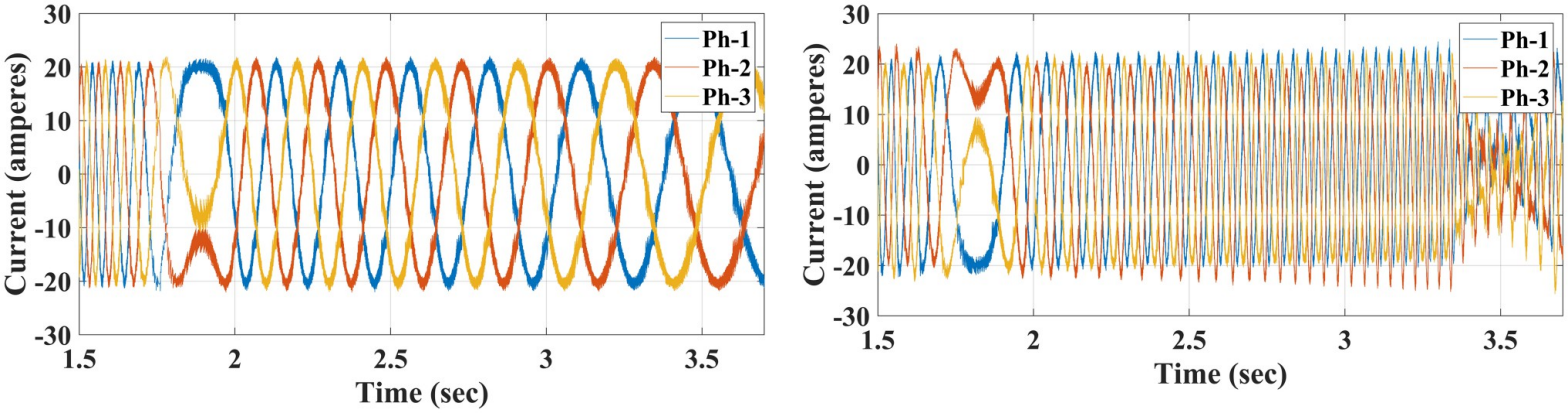
Current response of the motor at 20 Nm reverse load CCW for both Euler’s MPC (right) and HDT’s MPC (left).

**Fig 16:**
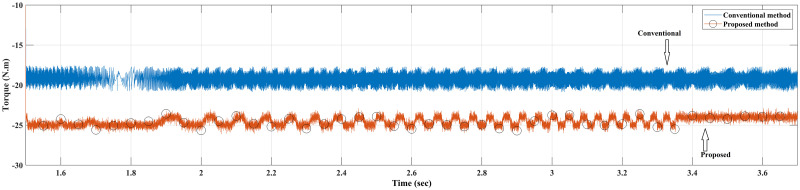
Torque response of the motor at 20 Nm reverse load CCW for Euler’s MPC and HDT’s MPC.

**Case IV**: When the motor is in CCW rotation with a 20 Nm load at *t* = 2 sec, the applied torque is higher than the motor-rated torque (20 Nm), and that applied load will stop the motor if the drawn current is limited to 20A. As a result, the conventional method is unable to maintain the desired speed and the motor stumbles under a load of 20 Nm, resulting in stalling. Contrary to this, considering the various proposed methods in this study, the motor continues to handle the applied load reasonably well with no degradation within the current limit of 20 A, although it fluctuates for a short time when it first applies the load before reaching the steady-state value. Interestingly, the proposed method had slightly higher current amplitude and frequency, which probably allows the proposed method to handle the high applied load while enhancing the modelling and prediction technique with better performance (Figs 14–16).

## 5 Conclusion

In this study, a new HDT is introduced to discretise prediction equations for MPFOC for better modelling accuracy and robust performance. The new technique is implemented for the three-phase IM model to enhance the IM output torque performance under varying load conditions. The PIL verification results show the effectiveness of the proposed DT in terms of response speed, torque ripple mitigation and enhanced load handling capability. The proposed method achieves a reduction of up to 20% torque ripple whereas, the load handling capability of the system has increased up to 10% under the proposed DT. Additionally, under varying loading conditions, the system gives 20% improvement in rise time and 23% settling time compared to its counterparts.
